# Undernutrition risk and obesity increase the risk of osteosarcopenia in Mexican adults aged 50 and over: a prospective cohort study

**DOI:** 10.3389/fnut.2024.1499453

**Published:** 2025-01-20

**Authors:** Isabel Omaña-Guzmán, Ashuin Kammar-García, Luis Miguel Gutierrez-Robledo, Oscar Rosas-Carrasco

**Affiliations:** ^1^Pediatric Obesity Clinic and Wellness Unit, Hospital General de México “Dr. Eduardo Liceaga”, Mexico City, Mexico; ^2^Dirección de Investigación, Instituto Nacional de Geriatría, Mexico City, Mexico; ^3^Geriatric Assessment Center, Health Department, Iberoamerican University, Mexico City, Mexico

**Keywords:** undernutrition risk, obesity, osteosarcopenia, adults, older adults, Mexico, cohort study

## Abstract

**Introduction:**

Undernutrition risk in adults is a common but undiagnosed condition, while obesity is highly prevalent in this population. Osteosarcopenia is the coexistence of sarcopenia and osteopenia/osteoporosis and is related to higher morbidity and mortality. Undernutrition has been identified as an associated factor of osteosarcopenia; however, it is unknown whether undernutrition risk is also related to this condition. On the other hand, obesity has been associated with osteosarcopenia, and several biological mechanisms in the relationship between muscle, bone, and fat have been identified. However, in both cases, there is a lack of longitudinal studies that allow evaluation of whether these conditions precede and increase the risk of osteosarcopenia. Therefore, the objective was to evaluate the association between undernutrition risk and obesity with osteosarcopenia among Mexican community-dwelling adults aged 50 and over.

**Methods:**

This is a secondary longitudinal study from the FraDySMex cohort. We considered data from 2014 and 2015 as baseline evaluations and 2019 as the follow-up evaluation. Undernutrition risk was assessed using the Mini Nutritional Assessment, obesity was assessed based on body fat percentage measured by DXA, and osteosarcopenia was diagnosed according to the FNIH criteria. To evaluate the association of obesity and undernutrition risk, we estimated mixed-effects logistic regression models. The final model was adjusted for sex, age, comorbidity, education, physical activity, and cognitive impairment.

**Results:**

A total of 304 participants with two evaluations (baseline and follow-up) were included in the study. The baseline mean age of participants was 69.6 years (SD 9.1), with ages ranging from 50 to 92 years. Most of the participants were female (83.2%), 40% had between 7 and 12 years of education, and almost half were categorized as sedentary (47.8%) at baseline evaluation. Both undernutrition risk and obesity increased the risk of osteosarcopenia, with an OR of 2.24 (95% CI: 1.20–4.19) and an OR of 2.22 (95% CI: 1.17–4.23), respectively.

**Conclusion:**

Our findings suggest that undernutrition risk, on the one hand, and obesity, on the other hand, can precede and increase the risk of osteosarcopenia in community-dwelling adults aged 50 and over.

## 1 Introduction

Aging is associated with a decline in the physiological functions of various organs and systems, which affect individuals’ abilities and increase their risk of developing diseases. These changes are influenced by environmental factors and individual characteristics, such as genetics and lifestyle. Nutritional status is an important factor that could impact the development of adverse outcomes associated with aging, both positively and negatively (1).

According to the World Health Organization (WHO), malnutrition includes both deficiencies and excesses of energy and/or nutrients. This can manifest as undernutrition, micronutrient deficiencies or excesses, and overweight/obesity (2). Indeed, an individual can experience both overweight/obesity and micronutrient deficiencies simultaneously, which is known as the double burden of malnutrition (3). Otherwise, clinical nutrition societies define malnutrition as synonymous with undernutrition (4–6). Overall, according to them, an undernutrition state is characterized by a low body mass index (BMI), weight loss, low fat-free mass or muscle mass, and low energy intake (4–6). In this study, we use the terms undernutrition and obesity as forms of malnutrition (2), recognizing that undernutrition risk precedes undernutrition (5). Undernutrition risk can be identified through validated nutritional screening tools (4, 7); its detection represents an opportunity to prevent undernutrition and its consequences. There is limited worldwide evidence about the magnitude of undernutrition risk in community-dwelling older adults. However, the scarce studies available have shown that it is a common and undiagnosed condition (1, 8). A meta-analysis (9) estimated a pooled prevalence of undernutrition risk at 26.5%. Conversely, obesity, characterized by an excess of adipose tissue, is a public health concern at all stages of life, including aging. In the United States (USA), the prevalence of obesity in older adults was 42.8% in 2017–2018 (10), while in Mexico, it was 37.1% in 2020–2023 (11). Another study conducted in the United States estimated obesity prevalence rates of 42% in men and 49% in women, based on body fat percentage measured using dual-energy X-ray absorptiometry (DXA) (12).

Body composition changes associated with aging may lead to phenotypes that increase the risk of various adverse outcomes. Sarcopenia is defined as a reduction in both the quantity and quality of muscle mass (13), while osteopenia/osteoporosis is characterized by low bone mineral density (BMD) and damage to bone microarchitecture (14). The coexistence of sarcopenia and osteopenia/osteoporosis, known as osteosarcopenia, is common in older adults. A meta-analysis (15) estimated the global prevalence of osteosarcopenia at 21%. This condition impairs functional capacity and increases the risk of adverse outcomes such as falls, fractures (14), frailty, and mortality (16).

Although undernutrition and obesity have been documented to be related to sarcopenia and osteoporosis as separate conditions in epidemiological studies, there has been limited focus on osteosarcopenia. Undernutrition has been reported as a risk factor for osteosarcopenia (16, 17); however, longitudinal studies are lacking, and the impact of undernutrition risk remains unexplored. Regarding obesity, although some authors have identified the osteosarcopenic obesity phenotype (18, 19), there is a dearth of studies evaluating whether obesity precedes and increases the risk of developing osteosarcopenia. Therefore, the objective of this study was to evaluate the association of undernutrition risk and obesity with osteosarcopenia among Mexican community-dwelling adults aged 50 and over.

## 2 Methods

### 2.1 Study design and population

This is a secondary analysis of the prospective cohort FraDySMex (Frailty, Dynapenia, and Sarcopenia in Mexican Adults). FraDySMex is carried out in community-dwelling adults aged 50 years or older living in Mexico City. The first evaluation wave was carried out in 2014 (*n* = 339), the second in 2015 (*n* = 491), and the third in 2019 (*n* = 852). The inclusion criteria for this study were (1) individuals who could move with or without assistive devices, (2) the ability to answer the study questionnaire independently or with the help of a caregiver, and (3) a total score of the Mini-Mental State Examination (MMSE) ≥10 points. Individuals with the following characteristics were excluded: (1) institutionalized, (2) decreased alertness, or (3) the presence of any acute or unstable chronic condition that could affect their ability to answer the proposed questionnaires or complete the objective evaluation. The 2014 and 2015 waves were carried out at the Functional Evaluation Research Laboratory at the National Institute of Geriatrics, while the 2019 wave was conducted at the National Institute of Geriatrics and the Geriatric Assessment Center at the Ibero American University.

**Figure 1:**
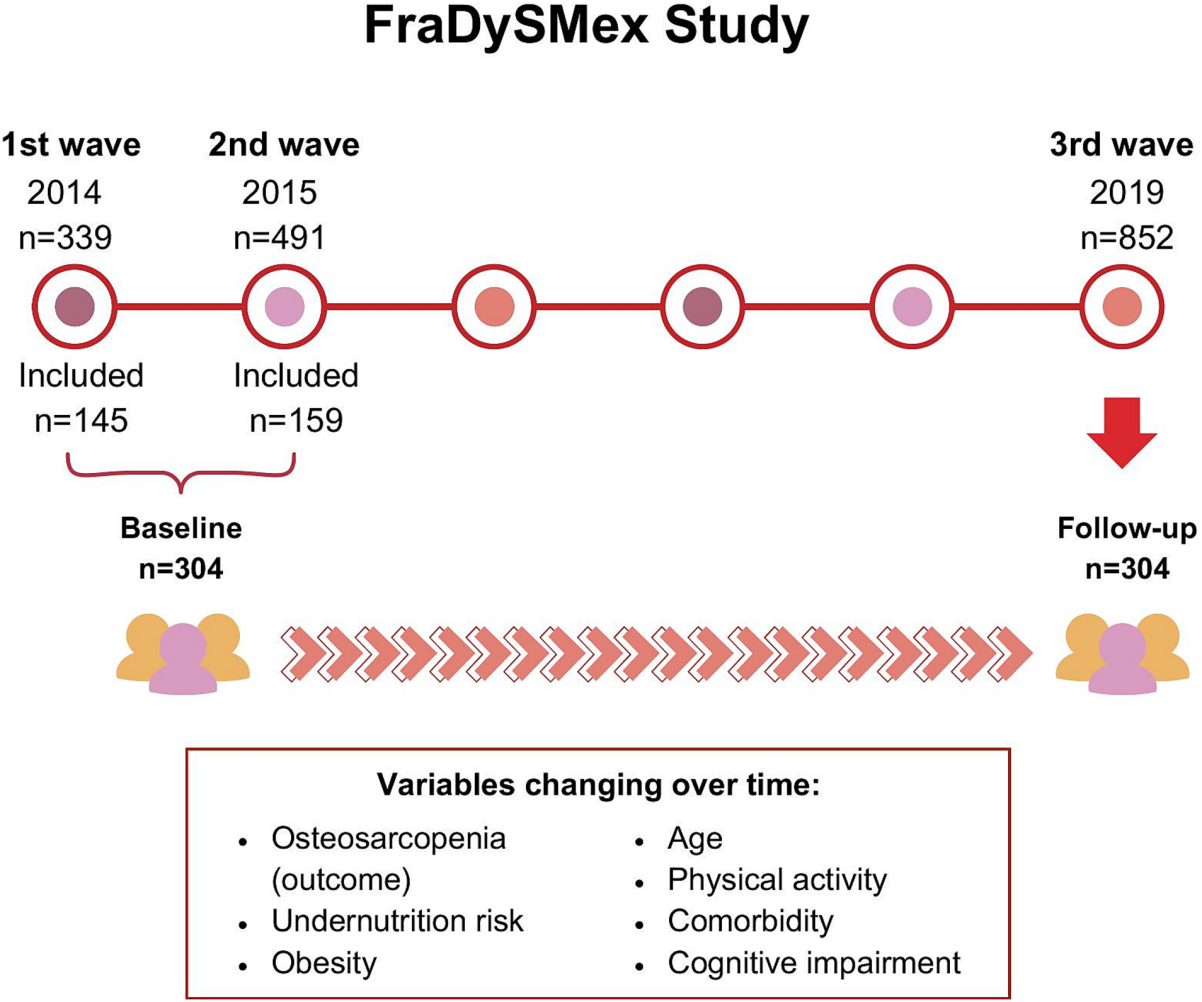
FraDySMex study design.

For the present study, we considered data from 2014 and 2015 as the baseline evaluation and 2019 as the follow-up evaluation. Individuals with complete evaluations of body composition and undernutrition risk at both the baseline and follow-up waves were included (Figure 1).

This study was conducted following the Declaration of Helsinki principles, and all participants signed an informed consent letter. The study was approved by the Research Ethics Committees of the Angeles Mocel General Hospital (2014–2015–2019) and registered by the National Geriatrics Institute (DI-PI-002/2014, DI-PI-009/2019).

### 2.2 Undernutrition risk

Undernutrition risk was assessed using the Mini Nutritional Assessment (MNA) (20). The MNA evaluates four parameters: anthropometric data (BMI and arm and calf circumferences), dietary intake, general assessment (e.g., mobility, weight loss, and neuropsychological problems), and self-perception (e.g., perception of nutritional status). This tool classifies individuals into three categories: well-nourished, at risk of undernutrition, and undernourished. Considering the low prevalence of undernutrition (3.0%) in the studied population, we decided to combine the categories into two groups: (1) well-nourished and (2) undernutrition/undernutrition risk. A total score of ≤23.5 was defined as undernutrition/undernutrition risk (20).

### 2.3 Obesity

Body composition was measured by DXA (Hologic Discovery-WI; Hologic, Bedford, MA). Obesity was defined as a fat percentage > 40 for women and > 30 for men (12).

### 2.4 Osteosarcopenia diagnosis

Osteosarcopenia was defined as the coexistence of osteopenia/osteoporosis and sarcopenia. Osteopenia/osteoporosis was diagnosed based on the BMD T-score of the right or left hip, using the higher of the two values as measured by DXA. If these values were unavailable (8% of the data), the total body BMD T-score was considered. The correlation between hip BMD and total body BMD measurements was good (*r* = 0.60). A T-score of ≤ −1.0 was used as the cut-off value, consistent with standard diagnostic criteria (21).

Sarcopenia was diagnosed using the FNIH criteria (22): (1) appendicular lean mass (ALM) adjusted for body mass index (BMI) (ALM_BMI_) <0.789 for men and < 0.512 for women and (2) handgrip strength was <26 kg for men and < 20 for woman. The FNIH criteria were applied because they were developed using studies that included Hispanic participants.

Hand grip strength was assessed using a hydraulic hand dynamometer (Jamar, Duluth, MN). Participants were seated in a chair with their elbows flexed at a 90-degree angle and their forearms resting on a table, ensuring proper support for the ulnar side. The wrist was positioned slightly extended (0–15 degrees) above the table surface (23). Each grip effort lasted between 5 and 10 s. Participants performed three trials, with a rest interval of 30–50 s between attempts. The highest value obtained from the dominant hand was recorded as the final measurement.

### 2.5 Sociodemographic and clinical variables

Sociodemographic data (age, sex, education, and marital status) were obtained through a questionnaire administered during each evaluation wave.

Physical performance and gait speed were assessed as part of the comprehensive clinical and functional evaluation. Physical performance was assessed using the Short Physical Performance Battery (SPPB) (24). Participants were categorized as having low physical performance if their total SPPB score was less than 8. Gait speed was measured during a 6-m walk at a usual pace using the GAITRite (Platinum 20) instrumented walkway (204 × 35.5 × 0.025 inches, sample rate 100 Hz). A gait speed of ≤0.7 m/s was used as the cut-off for identifying low gait speed (25).

Comorbidity was evaluated using the Charlson Index (26). A total score of <3 was considered low comorbidity, and a score of ≥3 was classified as high comorbidity. Cognitive impairment was assessed with the MMSE (27), using cut-off scores adjusted for education level: for individuals with 5 or more years of education, a total score ≤ 23; for those with 1–4 years of education, a score ≤ 19; and for those with less than 1 year of education, a total score ≤ 16. Physical activity was evaluated using the Community Healthy Activities Model Program for Seniors (CHAMPS) questionnaire (28) and categorized into two groups based on average weekly energy expenditure in METs from physical activities: (1) sedentary/low activity (<3 METs) and (2) moderate/vigorous activity (≥3 METs).

We evaluated alcohol intake as a categorical variable with the following categories: (1) no alcohol consumption, (2) alcohol intake of 14–28 g/day, less than once per week, and up to 7 days per week, and (3) alcohol intake exceeding 28 g/day, at least once per week, or up to 7 days per week. Smoking history was assessed by estimating the number of packs smoked per year and the total number of years each participant had smoked (29).

### 2.6 Statistical analysis

We estimated the frequencies and proportions of categorical variables to describe the studied population. Chi-squared tests were used for categorical variables, and Student’s *t*-tests were used for continuous variables, to compare baseline and follow-up differences between the osteosarcopenia and non-osteosarcopenia groups.

Given the longitudinal design of our study and the dynamic nature of both the outcome variable (osteosarcopenia) and the primary independent variables (obesity and undernutrition risk) over time, we used mixed-effects logistic regression models (MELRM) to evaluate their association. MELRM accounts for both between-subject and within-subject variability, providing a robust framework for analyzing repeated measures data.

The final model was adjusted for the following potential confounders: sex, age, comorbidities, education, physical activity, and cognitive impairment. Alcohol intake and smoking history were excluded from the final model as they did not significantly influence the risk of osteosarcopenia or affect the parameter estimates. Interaction terms between age group and the main independent variables (undernutrition risk and obesity) were tested to assess whether age influenced the association with osteosarcopenia. In addition, the interaction between undernutrition risk and obesity was evaluated to determine whether the simultaneous presence of these conditions increased the risk of osteosarcopenia. A *p*-value of <0.05 was considered statistically significant, and all analyses were performed using STATA 15.0.

## 3 Results

### 3.1 Baseline and follow-up characteristics of participants

A total of 304 participants were included in the study, all of whom underwent two evaluations: baseline and follow-up (Figure 1). Table 1 summarizes the baseline characteristics. The mean age of participants was 69.6 years (SD 9.1), with ages ranging from 50 to 92 years. Most participants were female (83.2%), 40% had 7–12 years of education, and nearly half were categorized as sedentary (47.8%). Obesity was prevalent in 60.5% of the participants, while 34.2% were at risk of undernutrition. The baseline prevalence of osteosarcopenia was 14.1% (*n* = 43).

**Table 1:** Baseline characteristics of participants and differences between the osteosarcopenia and non-osteosarcopenia groups.

Characteristics	Total *n* = 304	Non-osteosarcopenia *n* = 261	Osteosarcopenia *n* = 43	*p*-value
	*n* (%)	*n* (%)	*n* (%)	
Sex				
Female	253 (83.2)	214 (82.0)	39 (90.7)	0.157
Male	51 (16.8)	47 (18.0)	4 (9.3)	
Undernutrition risk (MNA)				
Well-nourished (≤23.5 points)	200 (65.8)	178 (68.2)	22 (51.2)	0.029
Undernutrition risk (≥24 points)	104 (34.2)	83 (31.8)	21 (48.8)	
Obesity				
No	120 (39.5)	106 (40.6)	14 (32.6)	0.317
Yes	184 (60.5)	155 (59.4)	29 (67.4)	
Age (years)				
50–59	37 (12.2)	36 (13.8)	1 (2.3)	0.005
60–69	109 (35.9)	99 (37.9)	10 (23.3)	
70–79	107 (35.2)	87 (33.3)	20 (46.5)	
≥80	51 (16.8)	39 (14.9)	12 (27.9)	
Education (years)				
≥13	74 (24.3)	64 (24.5)	10 (23.2)	0.402
7 a 12	149 (40.0)	131 (50.2)	18 (41.9)	
0 a 6	81 (26.6)	66 (25.3)	15 (34.9)	
Comorbidity (Charlson Index)				
Low (≤2 points)	276 (90.8)	237 (90.8)	39 (90.7)	0.982
High (≥3 points)	28 (9.2)	24 (9.2)	4 (9.3)	
Cognitive impairment (MMSE)				
No	275 (90.5)	238 (91.2)	37 (86.0)	0.288
Yes	29 (9.5)	23 (8.8)	6 (14.0)	
Physical activity				
Sedentary/low activity	219 (73.2)	187 (73.0)	32 (74.4)	0.851
Moderate/vigorous activity	80 (26.7)	69 (27.0)	11 (25.6)	
Physical performance (SPPB)				
Good (<8 points)	226 (74.3)	203 (77.8)	23 (53.5)	0.001
Low (≥8 points)	78 (25.7)	58 (22.2)	20 (46.5)	
Gait speed (meters/s)				
Good (>8)	246 (81.7)	219 (84.6)	27 (64.3)	0.002
Low (≤0.7)	55 (18.3)	40 (15.4)	15 (35.7)	
Alcohol intake				
No consumption	137 (45.7)	112 (44.7)	22 (51.2)	0.387
14–28 g/day, less than once per week, and up to 7 days per week	154 (51.3)	133 (51.8)	21 (48.8)	
≥29 g/day, at least once per week, or up to 7 days per week	9 (3)	9 (3.5)	0	

	Mean (SD)	Mean (SD)	Mean (SD)	*p*-value
Age (years)	69.6 (9.1)	68.8 (8.9)	74.5 (8.5)	0.0001
MNA score	24.5 (2.5)	24.6 (2.4)	23.9 (2.6)	0.121
Body fat (%)	39.6 (6.6)	39.4 (6.7)	41.1 (5.7)	0.113
BMI (kg/m^2^)	27.9 (4.4)	27.9 (4.3)	27.9 (5.2)	0.993
Weight (kg)	65.1 (11.9)	65.7 (11.6)	61.8 (13.3)	0.049
Height (m)	1.53 (0.1)	1.53 (0.08)	1.50 (0.06)	0.0004
Grip strength (kg)	20.4 (6.9)	21.6 (6.7)	13.4 (3.2)	<0.001
Appendicular lean mass (kg)	14.7 (3.4)	15.0 (3.5)	13.1 (2.7)	0.0005
ALM_BMI_	0.53 (0.1)	0.54 (0.1)	0.47 (0.08)	0.0005
BMD hip total T-score	−1.6 (1.5)	−1.5 (1.6)	−2.6 (0.8)	<0.001
Pack-years of smoking	4.4 (11.7)	4.6 (0.7)	3.0 (1.1)	0.411
Years of smoking	9.5 (15.1)	9.4 (14.8)	10.4 (2.6)	0.679

MNA, Mini Nutritional Assessment.

MMSE, Mini-Mental State Examination: cognitive impairment was considered if total score 17–23.5 and ≥ 4 years of study; total score ≤ 19 and 1–4 years of study; total score ≤ 16 and ≤ 1 of study.

ALM_BMI_, appendicular lean mass adjusted by body mass index.

BMD, bone mineral density.

SPPB, Short Physical Performance Battery.

Data compared by chi-squared and Student’s t-tests.

Significant baseline differences were observed between the osteosarcopenia and non-osteosarcopenia groups. A higher proportion of individuals in the osteosarcopenia group were at risk of undernutrition (48.8% vs. 31.8%, *p* = 0.029) and belonged to older age groups (70–79 years: 46.5% vs. 33.3%; ≥80 years: 27.9% vs. 14.9%, *p* = 0.005). While obesity was more common in the osteosarcopenia group compared to the non-osteosarcopenia group, this difference was not statistically significant. As expected, the osteosarcopenia group had a higher proportion of individuals with low physical performance (46.5% vs. 22.2%) and reduced gait speed (35.7% vs. 14.5%) compared to the non-osteosarcopenia group. No differences were found in BMI. However, the osteosarcopenia group showed significantly lower values for weight, height, muscle strength, and muscle mass indicators (Table 1).

In the follow-up evaluation (2019 wave), participants had a mean age of 73.8 years (SD = 9.3), with an age range of 53–97 years. The proportion of individuals at risk of undernutrition increased by 12.5 percentage points, while the prevalence of obesity decreased by 5.6 percentage points (Table 2). The prevalence of osteosarcopenia increased by 6.7 percentage points, reaching 21.1% (*n* = 64).

**Table 2:** Follow-up characteristics of participants and differences between the osteosarcopenia and non-osteosarcopenia groups.

Characteristics	Total *n* = 304	Non-osteosarcopenia *n* = 240	Osteosarcopenia *n* = 64	*p*-value
	*n* (%)	*n* (%)	*n* (%)	
Undernutrition risk (MNA)				
Well-nourished (≤23.5 points)	162 (53.3)	138 (57.5)	24 (37.5)	0.004
Undernutrition risk (≥24 points)	142 (46.7)	102 (42.5)	40 (62.5)	
Obesity				
No	137 (45.1)	115 (47.9)	22 (34.4)	0.053
Yes	167 (54.9)	125 (52.1)	42 (65.6)	
Sex				
Female	253 (83.2)	198 (82.5)	55 (85.9)	0.513
Male	51 (16.8)	42 (17.5)	9 (14.1)	
Age (years)				
50–59	26 (8.5)	24 (10.0)	2 (3.1)	<0.0001
60–69	79 (26.0)	73 (30.4)	6 (9.4)	
70–79	117 (38.5)	96 (40.0)	21 (32.8)	
≥80	82 (27.0)	47 (19.6)	35 (54.7)	
Education (years)				
≥13	74 (24.4)	67 (27.9)	7 (10.9)	0.001
7 a 12	149 (49.0)	119 (49.6)	30 (46.9)	
0 a 6	81 (26.6)	54 (22.5)	27 (42.2)	
Comorbidity (Charlson Index)				
Low (≤2 points)	265 (87.2)	213 (88.7)	52 (81.2)	0.111
High (≥3 points)	39 (12.8)	27 (11.3)	12 (18.8)	
Cognitive impairment (MMSE)				
No	271 (89.1)	221 (92.1)	50 (78.1)	0.001
Yes	33 (10.9)	19 (7.9)	14 (21.9)	
Physical activity				
Sedentary/low activity	82 (78.4)	180 (76.6)	52 (85.3)	0.144
Moderate/vigorous activity	64 (21.6)	55 (23.4)	9 (14.2)	
Physical performance (SPPB)				
Good (<8 points)	215 (70.7)	186 (77.5)	29 (45.3)	<0.001
Low (≥8 points)	89 (29.3)	54 (22.5)	35 (54.7)	
Gait speed (meters/s)				
Good (>8)	205 (68.1)	181 (75.7)	24 (38.7)	<0.001
Low (≤0.7)	96 (31.9)	58 (24.3)	38 (61.3)	
Alcohol intake				
No consumption	112 (37.0)	83 (43.6)	29 (46.0)	0.201
14–28 g/day, less than once per week, and up to 7 days per week	179 (59.0)	148 (61.7)	31 (49.2)	
≥ 29 g/day, at least once per week, or up to 7 days per week	12 (4.0)	9 (3.7)	3 (4.8)	

	Mean (SD)	Mean (SD)	Mean (SD)	*p*-value
Age (years)	73.8 (9.3)	72.1 (8.6)	80.2 (9.1)	<0.0001
MNA score	23.6 (2.3)	23.8 (2.9)	22.6 (3.0)	0.003
Body fat (%)	38.5 (6.3)	38.04 (6.3)	40.2 (6.1)	0.014
BMI (kg/m^2^)	27.2 (4.7)	27.3 (4.8)	27.0 (4.6)	0.710
Weight (kg)	63.1 (12.2)	64.3 (12.1)	58.6 (11.5)	<0.0001
Height (m)	1.52 (0.08)	1.53 (0.08)	1.47 (0.07)	<0.0001
Grip strength (kg)	18.5 (7.0)	20 (6.8)	12.9 (4.0)	<0.0001
Appendicular lean mass (kg)	14.5 (3.2)	14.9 (3.2)	12.7 (2.5)	<0.0001
ALM_BMI_	0.54 (0.1)	0.55 (0.1)	0.48 (0.1)	<0.0001
BMD hip total T-score	−1.4 (1.1)	−1.2 (1.1)	−2.1 (0.8)	<0.0001
Pack-years of smoking	4.5 (11.7)	5.0 (0.8)	2.4 (0.7)	0.113
Years of smoking	10.0 (15.9)	10.7 (1.1)	7.5 (1.7)	0.151

MNA, Mini Nutritional Assessment.

MMSE, Mini-Mental State Examination: cognitive impairment was considered if total score 17–23.5 and ≥ 4 years of study; total score ≤ 19 and 1–4 years of study; total score ≤ 16 and ≤ 1 of study.

ALM_BMI_, appendicular lean mass adjusted by body mass index.

BMD, bone mineral density.

SPPB, Short Physical Performance Battery.

Data compared by chi-squared and Student’s t-tests.

When comparing the groups, the proportion of individuals at risk of undernutrition remained higher in the osteosarcopenia group (62.5% vs. 42.5%, *p* = 0.004), as did the proportion of older individuals (≥80 years: 54.7% vs. 19.6%, *p* < 0.001). Furthermore, the osteosarcopenia group had a higher proportion of individuals with lower education levels (42.2% vs. 22.5%, *p* = 0.001) and cognitive impairment (21.9% vs. 7.9%, p = 0.001). In this wave, differences in the MNA total score and body fat percentage were observed, with the osteosarcopenia group showing lower MNA scores and higher fat mass. Notably, BMI was the only variable that did not differ significantly between the two groups (Table 2).

For the final model, since there were relatively fewer individuals in the age group of 50–59 years old (12.2 and 8.5% for the baseline and follow-up evaluations), we combined this group with the 60–69 years old category. We confirmed that the parameter estimation remained consistent after this re-grouping.

### 3.2 Association of undernutrition risk and obesity with osteosarcopenia

Both undernutrition risk and obesity independently increased the risk of osteosarcopenia to a similar extent, as shown in Table 3 (OR = 2.24, 95% CI 1.20–4.19 and OR = 2.22, 95% CI 1.17–4.23, respectively). At baseline, 29.9% of participants had both obesity and undernutrition risk, and this proportion increased to 39.5% at follow-up. To assess whether the simultaneous presence of these conditions amplified the risk of osteosarcopenia, an interaction between these two variables was tested. However, the interaction was not significant, suggesting that the effects of obesity and undernutrition risk on osteosarcopenia are independent. In addition, no significant interaction was found between age group and the risk of undernutrition or obesity.

**Table 3:** Impact of undernutrition risk and obesity on osteosarcopenia: a longitudinal analysis.

	OR	*p*-value	CI 95%
Undernutrition risk (MNA)			
Well–nourished (≤23.5 points)	REF	–	–
Undernutrition risk (≥24 points)	2.24	0.011	1.20–4.19
Obesity			
No	REF	–	–
Yes	2.22	0.015	1.17–4.23
Sex			
Females	REF	–	–
Male	0.58	0.230	0.24–1.41
Age (years)			
50–69	REF	–	–
70–79	2.52	0.015	1.19–5.30
≥80	7.64	<0.001	3.28–17.78
Education (years)			
≥13	REF	–	–
7–12	1.17	0.699	0.52–2.62
0–6	1.72	0.242	0.69–4.29
Comorbidity			
Low (≤2 points)	REF	–	–
High (≥3 points)	1.96	0.130	0.82–4.68
Physical activity			
Moderate/vigorous activity	REF	–	–
Sedentary/Low activity	1.17	0.619	0.62–2.22
Cognitive impairment (MMSE)			
No	REF	–	–
Yes	1.49	0.336	0.66–3.34

REF, reference group category.

MMSE, Mini-Mental State Examination: If total score 17–23.5 and ≥ 4 years of study; total score ≤ 19 and 1–4 years of study; total score ≤ 16 and ≤ 1 of study. Obesity (total fat by DXA > 40% for women and > 30% for men).

Model adjusted for undernutrition risk, obesity, sex, age, education, comorbidities, physical activity, and cognitive impairment.

Age was another significant risk factor for osteosarcopenia. Individuals aged 70–79 had an increased risk of osteosarcopenia (OR = 2.52, 95% CI: 1.19–5.30) compared to those aged 50 to 69, but the risk increased substantially for those aged 80 and over (OR = 7.64, 95% CI: 3.28–17.78).

## 4 Discussion

Both undernutrition risk and obesity increased the risk of osteosarcopenia in Mexican community-dwelling adults aged 50 and over. Considering the longitudinal design and the statistical analyses used, these findings suggest that, on the one hand, undernutrition risk and, on the other hand, obesity can precede and increase the risk of osteosarcopenia.

The pathophysiology of osteosarcopenia involves several mechanisms that disrupt the crosstalk between muscle and bone (30). Muscle and bone secrete myokines (e.g., myostatin) and osteokines (e.g., osteocalcin), respectively, which regulate the biological functions of these tissues and play a role in the muscle–bone crosstalk. Dysregulation in these myokines and osteokines contributes to the development of osteosarcopenia (31).

Undernutrition has been identified as a factor associated with osteosarcopenia in cross-sectional studies. However, the impact of the undernutrition risk had not been assessed longitudinally. Our research showed that this condition significantly increased the risk of osteosarcopenia (OR = 2.24) in a similar magnitude as a meta-analysis (16) estimated for undernutrition (OR = 2.35). Undernutrition involves muscle mass loss and deficiencies in protein, energy, and micronutrients, particularly vitamin D and calcium, contributing to the development of sarcopenia and osteopenia/osteoporosis (32–34) and consequently osteosarcopenia. However, we identified that undernutrition risk also contributes to this condition. This is highly relevant because, as our findings have shown, undernutrition risk is prevalent among adults aged 50 and older living in the community. Nonetheless, this alteration in nutritional status often remains undiagnosed in the general population (32). Overall, undernutrition has been associated with increased morbidity and mortality. Therefore, individuals identified as at risk of this condition should undergo thorough evaluation (5).

On the other hand, obesity also increases the risk of osteosarcopenia to a similar extent as the undernutrition risk (OR = 2.22). No studies were identified that evaluated the association between obesity and the risk of developing osteosarcopenia. Most of the evidence has focused on studying the association between obesity and osteoporosis. Previous studies suggested that obesity decreased the risk of osteoporosis. However, recent evidence indicates that it increases the risk (35, 36).

While the coexistence of obesity, sarcopenia, and osteoporosis has been documented, and some biological mechanisms underlying muscle–bone–fat interactions have been identified, there remains a lack of longitudinal studies assessing whether obesity precedes and increases the risk of muscle–bone alterations, leading to osteosarcopenia. Therefore, our findings provide epidemiological evidence supporting the idea that an excess of fat mass may precede and increase the risk of developing osteosarcopenia.

**Figure 2:**
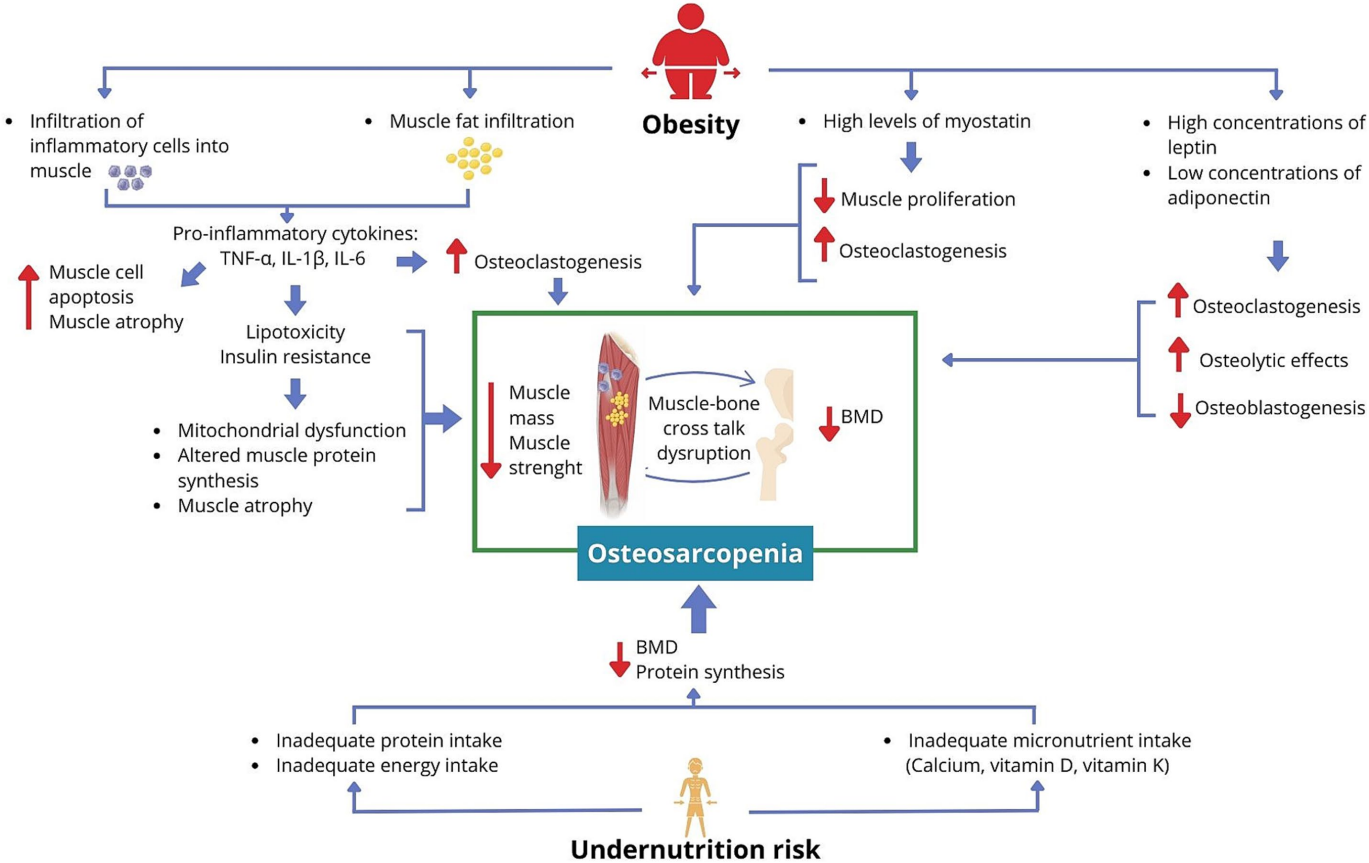
Proposed mechanism linking obesity and undernutrition risk to increased osteosarcopenia risk.

Adipose tissue can disrupt the crosstalk between muscle and bone (14) through inflammatory processes, lipotoxicity, and endocrine factors (37). Obesity is associated with an inflammatory status characterized by high concentrations of inflammatory cytokines such as IL-6 and TNF-*α*, which can affect bone and muscle tissues (35, 37, 38). This pro-inflammatory state promotes the infiltration of inflammatory cells (e.g., macrophages) into muscle tissue, where these cells secrete TNF-α, IL-1β, and IL-6, which increase muscle cell apoptosis and promote muscle atrophy (38, 39). At the same time, TNF-α and IL-6 negatively impact BMD by promoting the preferential differentiation of osteogenic cells into osteoclasts, leading to bone resorption effects (35, 37). Furthermore, adiposity promotes fat infiltration into non-adipocyte cells, such as myocytes. This contributes to increased inflammatory cytokines, lipotoxicity, and insulin resistance, which are associated with mitochondrial dysfunction, impaired muscle protein synthesis, and muscle atrophy (38, 40). Obesity is also associated with elevated leptin levels, which have been linked to both osteogenic and osteolytic effects in animal models (38). In addition, obesity is characterized by low adiponectin levels, an adipokine that promotes osteoblastogenesis, inhibits osteoclastogenesis (37, 41), and prevents inflammation and fat infiltration in muscle tissue (38). Consequently, low adiponectin levels can negatively impact both BMD and muscle mass. Furthermore, obesity is associated with increased levels of myostatin (42), a myokine that suppresses muscle growth and promotes osteoclastogenesis, thereby adversely affecting both muscle and bone tissues (43). The biological mechanisms linking muscle, bone, and adipose tissue, summarized in Figure 2, illustrate how obesity may contribute to an increased risk of osteosarcopenia.

Consistent with the scientific evidence (15, 16), age was recognized as the main risk factor for osteosarcopenia. The risk increases in the 70- to 79-year age group (OR = 2.52) and becomes even higher in those aged 80 and over.

This study has some limitations that should be considered. The lack of a probabilistic and representative sample limits the extrapolation of the results to other populations in Mexico and other countries. In addition, we could not evaluate the intake of energy, macronutrients, and micronutrients, which could have expanded the nutritional status assessment. On the other hand, we did not measure biomarkers related to obesity or muscle and bone physiology, such as adipokines, myokines, osteokines, growth factors, and cytokines, which could have broadened the associations observed. However, the study has methodological strengths. First, the variables were evaluated using high-quality tools, including DXA, and validated scales such as the MNA. Moreover, the longitudinal design and the statistical analysis suggest a causal relationship between undernutrition risk, obesity, and osteosarcopenia.

## 5 Conclusion

Undernutrition risk and obesity increase the risk of osteosarcopenia in community-dwelling adults aged 50 and over. Given the global epidemiological context where low- and middle-income countries are experiencing the double burden of malnutrition (both undernutrition and obesity) at various life stages, and considering that osteosarcopenia is prevalent among older adults, these findings underscore the urgent need to address both undernutrition and obesity in adults beginning at age 50. These findings have significant implications for healthcare in all clinical settings, where routine nutritional assessments should be conducted to identify adults at nutritional risk, including undernutrition or obesity. Furthermore, it is crucial to design and implement health programs aimed at diagnosing, preventing, and treating these nutritional alterations to prevent the development of osteosarcopenia and its adverse outcomes in a timely manner.

## Data Availability

The datasets presented in this article are not readily available because the datasets generated and/or analyzed during the current study are not publicly available due to participants did not explicitly consent to share their data on a public site, but data is available from the corresponding author on reasonable request. Requests to access the datasets should be directed to oscar_rosas_c@hotmail.com.
